# An inter- laboratory proficiency testing exercise for rabies diagnosis in Latin America and the Caribbean

**DOI:** 10.1371/journal.pntd.0005427

**Published:** 2017-04-03

**Authors:** Alfonso Clavijo, Mary H. Freire de Carvalho, Lillian A. Orciari, Andres Velasco-Villa, James A. Ellison, Lauren Greenberg, Pamela A. Yager, Douglas B. Green, Marco A. Vigilato, Ottorino Cosivi, Victor J. Del Rio-Vilas

**Affiliations:** 1 Pan American Foot-and-Mouth Disease Center, Pan American Health Organization, Rio de Janeiro, Brazil; 2 National Center for Emerging and Zoonotic Infectious Diseases, Centers for Disease Control and Prevention, Atlanta, Georgia, United States of America; Colorado State University, UNITED STATES

## Abstract

The direct fluorescent antibody test (DFA), is performed in all rabies reference laboratories across Latin America and the Caribbean (LAC). Despite DFA being a critical capacity in the control of rabies, there is not a standardized protocol in the region. We describe the results of the first inter-laboratory proficiency exercise of national rabies laboratories in LAC countries as part of the regional efforts towards dog-maintained rabies elimination in the American region. Twenty three laboratories affiliated to the Ministries of Health and Ministries of Agriculture participated in this exercise. In addition, the laboratories completed an online questionnaire to assess laboratory practices. Answers to the online questionnaire indicated large variability in the laboratories throughput, equipment used, protocols availability, quality control standards and biosafety requirements. Our results will inform actions to improve and harmonize laboratory rabies capacities across LAC in support for the regional efforts towards elimination of dog-maintained rabies.

## Author summary

The incidence of dog-maintained rabies in Latin America has showed a significant reduction in the last 30 years. Although several countries have achieved its elimination there are still focalized areas in the region where rabies remain endemic. Many factors, including poverty, geographic and environmental features, as well as social, cultural, political and economic variables, have limited the effective implementation of canine rabies control programs in these endemic areas. Critical to the goal of control and elimination of rabies is a robust laboratory diagnostic capacity. This report presents the results of an Inter-laboratory proficiency testing exercise for rabies diagnosis by direct fluorescent antibody test among laboratories across Latin America and the Caribbean (LAC). Our results indicate that although all laboratories can perform the direct fluorescent antibody test, there are substantial differences in the overall results and test interpretation. This study identified important gaps in standardization and/or harmonization between laboratories which could be overcome and corrected with appropriate DFA protocols standardized across the LAC, including its broad distribution and proper training.

## Introduction

Conclusive rabies diagnosis can only be achieved by appropriate laboratory testing. Clinical and epidemiological diagnosis is challenging and leads to under-reporting [1, 2, 3]. The Direct Fluorescent Antibody test (DFA) for detection of rabies virus antigen remains as the gold standard test for laboratory diagnosis of rabies in post-mortem brain tissues [3].

In addition to its essential role in the confirmation of clinical rabies, laboratory diagnosis supports rabies surveillance and the clinical management of patients exposed to a potentially rabid animal. In the case of disease surveillance in rabies endemic countries, laboratory diagnosis of suspected rabid animals is important for assessing the distribution and prevalence of the disease in its major reservoir hosts. In the case of human exposures to potentially rabid animals, the decision to administer post-exposure prophylaxis (PEP) depends on the results of observation and/or laboratory testing of the animal involved [2]. Laboratory diagnosis can avert the financial losses incurred by the unnecessary application of PEP in the case of negative results, and trigger adequate human case patient management and adequate occupational health risk assessments in the case of positive results [4, 5].

Due to a concerted elimination effort by the Latin America and Caribbean (LAC) countries, cases of human rabies transmitted by dogs have been reduced to a small number in well-defined areas in the region [6, 7]. As part of the regional strategies towards dog-associated rabies elimination, it is necessary to standardize rabies diagnostic testing across LAC to improve quality, enhance laboratory diagnostic capability and allow multinational inter-laboratory comparisons and analyses of regional surveillance data [8, 9]. To address this need, the Action Plan for the Elimination of Dog-Transmitted Rabies in LAC [6] seeks the implementation of an Inter-American Network of Rabies Diagnostic Laboratories (REDILAR- from the Spanish acronym, Red Interamericana de Laboratorios de Diagnostico de Rabia) to facilitate rapid and reliable diagnosis, promote regional training opportunities and develop a regional laboratory quality assurance program. To this effect, and following recommendations from the 14th Regional Meeting of National Rabies Program Managers, (REDIPRA 14 from the Spanish acronym) [10] a proficiency testing exercise was carried out within the LAC countries. This report describes an Inter-laboratory proficiency testing (IPT) exercise for rabies diagnosis by DFA, and a baseline assessment of laboratory practices and infrastructure among rabies diagnostic laboratories in LAC. Specifically, the objectives of this IPT were to assess the diagnostic capacity of each laboratory to perform the DFA test and identify specific laboratory needs for training and equipment. In regards the assessment laboratory practices, the objective was to identify differences in the laboratory protocols that could explain discrepant laboratory results and provide baseline knowledge for regional standardization of protocols.

## Materials and methods

### Participating laboratories

Thirty national or regional (as in regions or states within the countries) rabies reference laboratories, affiliated with the ministries of health (MoH) and/or agriculture (MoAg) agreed to participate in the exercise in response to an invitation from the Pan American Foot-and-Mouth Disease Center (PANAFTOSA in Spanish) of the Pan American Health Organization (PAHO) located in Rio de Janeiro, Brazil. By targeting MoH and MoAg affiliated laboratories, the exercise aimed to comprehensively capture rabies diagnosis practices, on human and animal samples. Across LAC countries, MoH laboratories mostly perform diagnosis on human and domestic animals samples, whereas MoAg laboratories focus on livestock and wildlife submissions. Participation in the IPT exercise and baseline operations assessment was voluntary. Thirteen laboratories were affiliated with the MoH and seventeen with the MoAg. The 30 participating laboratories were located in the following countries: Argentina (MOH/MoAg), Bolivia (MOH/MoAg), Brazil (MOH/MoAg), Canada (MoAg), Chile (MOH), Colombia (MOH/MoAg), Costa Rica (MoAg), Cuba (MoAg), Ecuador (MoAg), Dominican Republic (MoAg), El Salvador (MoAg), Guatemala (MOH/MoAg), Haiti (MoAg), Honduras (MoAg), Mexico (MOH/MoAg), Nicaragua (MOH), Panama (MoAg), Paraguay (MOH/MoAg), Peru (MOH), Trinidad and Tobago (MoAg) and Uruguay(MoAg). The laboratories were randomly coded as L1 to L30.

### Source of panel material and panel composition

Brain tissue impression slides used in the exercise were prepared at the Poxvirus and Rabies Branch of the US Centers for Disease Control and Prevention (CDC) and represented samples received for routine rabies diagnosis or typing with variations in antigen load and distribution. All tissues were obtained from naturally occurring rabies cases in major host reservoir species without passage in a secondary susceptible animal. No animals were used or harmed in any way for this study.

Each PT panel constituted by 20 samples included 17 test samples and 3 controls. Positive tissues contained major rabies virus variants circulating in the Americas. Negative samples, encompassed tissues demonstrating complete absence of rabies virus antigen and artifacts, and some with atypical golden lipofuscin and atypical nonspecific fluorescent bacteria or fluorescent artifacts. Lipofuscin within CNS tissues is commonly detected in cerebellum of cats and brain tissues from older animals, and appears as golden "pseudo-inclusions" with diffuse outline when DFA slides are observed with FITC filters exciting in blue light wavelengths. The lipofuscin will be observed in tissues which are stained by immunofluorescence as well as in tissues without conjugate applied. Often inexperienced individuals reading slides, particularly those working with conjugates at less than optimal dilution or deficient microscope equipment, may not be able to observe rabies positive virus antigen as sparkling 4+ apple-green fluorescence, which in turn prevents distinguishing artifacts.

The samples with lipofuscin were purposely chosen among CDC archived samples to demonstrate the golden atypical objects easily distinguished by competent laboratorians.

Details of the rabies virus variants included in the IPT panel are listed in Table 1 by both predominant host reservoir species, and antigenic type as indicated by the use of a panel of eight monoclonal antibodies (Mabs) directed to the rabies virus N protein [11].

**Table 1:** Rabies panel composition and rabies virus variants used in this study.

ID	SAMPLE	AG VARIANT	PASSAGED ON	ORIGIN	COMMENTS	INTENSITY/
						AG DISTRIBUTION
**1**	Strong Positive Control	(Dog Variant)	Not Passaged	*Canis lupus familiaris*	Naturally infected animal	4+/4+
**2**	Weak Positive Control	3	Not Passaged	*Bos taurus*	Naturally infected	4+/2+
**3**	Negative Control	N/A	N/A	Negative healthy (Sheep)	Non-rabid animal	0/0
**4**	PT 1	1 (Dog Variant)	Not Passaged	*Canis lupus familiaris*	Brain stem or cerebellum	4+/3-4+
**5**	PT 2	(Negative)	N/A	*Felis catus*	Cat cerebellum containing lipofuschin	0/0 Golden autofluorescence
**6**	PT 3	7 (AZ Gray Fox)	Not passaged	*Urocyon cinereoargenteus*	Brain stem or cerebellum	4+/2-3+
**7**	PT 4	Negative Dog	N/A	*Canis lupus familiaris*	Very clear negative	0/0
**8**	PT 5	3 (Vampire)	Bovine	*Bos taurus*	Brain stem or Cerebellum	4+/2+
**9**	PT 6	4 (Tadarida brasiliensis)	Not Passaged	*Tadarida brasiliensis*	Brain from naturally infected	4+/3+
**10**	PT 7	(Negative)	N/A	*fox Infected with Listeria monocytogenes*	brain tissue containing bacteria	Non-specific staining
**11**	PT 8	Negative skunk	NA	*Mephitis mephitis*	DFA Negative sample	0/0
**12**	PT 9	South Central US North Central MX	Not Passaged	*Mephitis mephitis*	Brain stem or Cerebellum	4+/2+
**13**	PT 10	6 *(Lasiurus cinereus)*	Fox	*Lasiurus cinereus*	Brain from naturally infected	4+/4+
**14**	PT 11	Negative	Non-rabid	*Molossus molossus*	Non rabid animal	0/0
**15**	PT 12	*Western Eptesicus fuscus*	Not passaged	*Eptesicus fuscus*	Brain from naturally infected	4+/3-4+
**16**	PT 13	Negative	Non-rabid	*Ursus arctos*	Brain stem or cerebellum	0/0 Golden autofluorescence
**17**	PT 14	Negative	Non-rabid	*Sus domesticus*	Brain stem or cerebellum	Non-specific staining
**18**	PT 15	Negative	Non-rabid	*mouse*	*Sample infected with group G streptococcus*	0/0
**19**	PT 16	*Mongoose (PuertoRico)*	Not passaged	*Herpestes javanicus*	Brain stem or cerebellum	4+/4+
**20**	PT 17	Negative	Non-passaged	*Sheep*	Brain stem or Cerebellum	0/0 Negative

All rabies positive brain tissues were inactivated via gamma-irradiation with 5 x 10^6^ rads using a Gamma Cell Irradiator (Gammacell 220 Excel, MDS Nordion, Canada) to exclude any infectious agent in order to facilitate safe importation procedures. To confirm complete inactivation of rabies virus, isolation was attempted in mouse neuroblastoma (MNA) cell culture. Brain homogenates (10% w/v) were prepared in Magna NA Lyser tissue homogenizer tubes containing 1.4-mm (diameter) ceramic beads (Roche Applied Science, Penzberg, Germany), using 1.0 mL of MEM-10 as a diluent. For virus recovery, 100μL of test inoculum was added to 1mL of MEM-10 containing 5x10^6^ mouse neuroblastoma cells (MNA) in a T-25 tissue culture flask (Corning, NY). Tissue culture flasks were incubated at 0.5% CO_2_ at 37°C for 72 hours prior to passaging. All cultures were sub-passaged a minimum of three times. For infectivity assessments, Teflon-coated four well slides were seeded with 30uL of MEM-10 containing 0.5 x 10^6^ cells per mL, and incubated in a humid chamber at 0.5% CO_2_ at 37°C for 24 hours. The slides were then rinsed with phosphate-buffered saline (PBS), and fixed in cold acetone at -20°C for one hour. Cells were checked for the presence of RABV antigens by the DFA test, using optimal working dilutions of FITC-labeled anti-RABV mAb conjugate (Fujirebio Diagnostics, Inc., Malvern, PA, USA) after each passage. This testing method was performed in duplicate by multiple, qualified testing personnel at the CDC in Atlanta, Georgia.

After tissue inactivation and safety testing procedures concluded, tissues were re-tested by DFA to confirm antigen was not damaged. From all selected inactivated tissues, a total of 600 touch impression slides were prepared according to the standard protocol for the postmortem diagnosis of rabies by the DFA test [12]. All slides were acetone fixed at -20°C overnight and stored at -80°C until packaging and shipment from CDC to PANAFTOSA. After the panels arrived to PANAFTOSA in Brazil, the material was distributed to all the participating laboratories. Each shipment contained a document with instructions, and the results reporting sheet. The shipping time from dispatch to receipt by the laboratories was recorded. Laboratories were requested to submit the reporting sheet within two months of receipt of the samples.

### Panel testing

The slides were designed to cover different situations faced during routine testing in the participating laboratories. The participants were asked to test the samples using the standard protocol in their laboratory, record their results (positivity, intensity, and distribution of the fluorescence staining) in the reporting sheet, and the microscopic condition and impression quality of the tissues (Good, Acceptable, or Deficient) as evaluated by the laboratory operator. The microscopic condition refers to the integrity of the cells facilitating fluorescence interpretation (cytoplasmic or nuclear) under the microscope and the tissue impression quality refers to how well the tissue impressions were made and if there was sufficient material (tissue) to read the slide and reach a diagnosis as assessed by an overall visual inspection.

In addition, for the laboratory practices assessment, a questionnaire to study the variability of diagnostic techniques, available resources, quality control, and safety procedures was sent electronically to the participating laboratories in parallel to the panel

### Data synthesis and analysis

The sensitivity, specificity, and Cohen's kappa coefficient to measure the inter-rater agreement values were calculated for each laboratory. Samples for which no result was returned were considered missing and were not included in the analysis. Inconclusive samples (as classified by the laboratory on the reporting sheet) were excluded from the sensitivity and specificity calculations, but were considered for the kappa and concordance calculations. The level of concordance was calculated by laboratory and by sample as the number of results in agreement with those of the CDC over the total number of results; inconclusive results were included in the denominator.

Exploratory data analyses were carried out to investigate the relationship between the laboratories concordance results and laboratory practices as captured by the laboratory practices assessment. One-way ANOVA and Mann-Whitney U tests, the latter if the distributional assumptions underlying the parametric test were not satisfied, were used to compare groups of respondents to the questionnaire. An alpha of < 0.05 was considered statistically significant.

The overall microscopic condition and impression quality of the tissues for each laboratory panel and for each of the samples across the laboratories was calculated as the percent samples reported as deficient. In the case of two samples for which the operators from the same laboratory evaluated the quality of a slide differently (i.e. Good/Deficient or Adequate/Deficient), the quality was coded as Deficient for the purposes of the analysis. The data analysis was performed in R (i386 3.1.2) [13].

### Quality control verification

To assess potential shipping problems during transportation of PT panels to all participating laboratories, the stability of rabies antigen and reproducibility of the DFA test results was determined *a priori*. Two complete panel sets were maintained under 5 different temperatures (-80°C, -20°C and 4°C, room temperature and 37°C) as well as 3 different storage time (3, 7 and 14 days) to mimic potential transport and storage conditions. Thus, a great total of 600 slides with two tissue impressions were tested for all combinations of these conditions. At all time points and storage conditions one complete panel set was run using FDI Fujirebio Diagnostics Inc. cat# 800–092 and the other with EMD Millipore Light Diagnostics cat#5100). A control conjugate EMD Millipore Cat#5102 was used (non-rabies FITC labeled antibody of the same isotype as the FITC labelled monoclonal) to assess the specificity of the reaction. Both the fluorescent intensity and the antigen distribution were reported for each case. A total of 36 test results were reported and analyzed for each of the 20 samples in each panel tested. The result of this testing was compared against the baseline value provided by the CDC which was the result of samples stored at -80°C. There were sufficient sample slides for all planned storage variations except sample# 12 (WEF variant) whose tissue was very limited in amount available due to the small size of a bat brain.

## Results

### Direct fluorescent antibody test panel testing results

Of the 30 laboratories, from 21 countries, that accepted the invitation to participate in the exercise, 23 laboratories (9 affiliated to MoH and 14 affiliated to MoAg), from 18 countries, received the panel and completed the proficiency testing exercise. Seven laboratories, two in Argentina, two in Guatemala, one in Cuba, one in El Salvador and one in Paraguay, did not receive their samples due to shipping or customs problems. All the participating laboratories reported their results within two months after receiving the panel.

**Fig 1:**
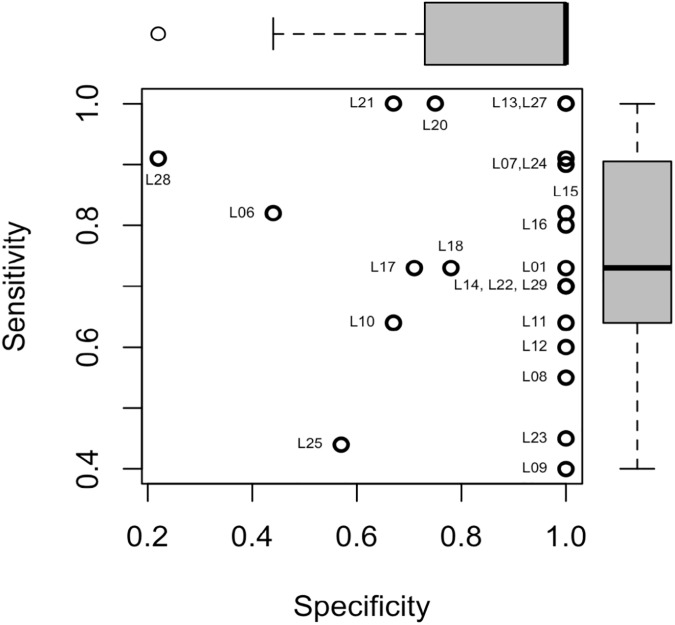
Scatterplot of Sensitivity by Specificity. The sensitivity (y-axis), or the true positive rates and the specificity (x-axis), or the true negative rates, for each laboratory are shown with an open circle. A laboratory correctly identifying all samples would be found in the upper right corner.

The agreement between the laboratory results and those of the CDC, as measured by the sensitivity, specificity, concordance and kappa values are shown in Table 2. Two laboratories correctly identified all samples tested (sensitivity and specificity of 1.0). However, 30% (7/23) of all laboratories reported at least one false positive and 83% (19/23) of all laboratories reported at least one false negative sample. The average sensitivity was 76% with a range of 40% to 100%. The average specificity was 88% with a range of 22% to 100%. While a majority of the laboratories had low false positive rates, there were considerable differences in the sensitivity (Fig 1). The mean concordance was 81% with a range of 50% to 100% and the mean kappa score was 0.56 with a range of 0.02 to 1.00.

**Table 2:** Results by Laboratory and agreement relative to CDC expected results.

Laboratory	Missing	Inconclusive	a	b	c	d	Sensitivity	Specificity	Concordance	Kappa
L01	0	0	8	0	3	9	0.73	1.00	0.85	0.71
L06	0	0	9	5	2	4	0.82	0.44	0.65	0.27
L07	0	2	9	0	1	8	0.90	1.00	0.94	0.73
L08	0	2	6	0	5	7	0.55	1.00	0.72	0.38
L09	1	0	4	0	6	9	0.40	1.00	0.68	0.39
L10	0	0	10	0	1	9	0.91	1.00	0.95	0.90
L11	0	0	7	0	4	9	0.64	1.00	0.80	0.61
L12	0	1	6	0	4	9	0.60	1.00	0.79	0.54
L13	0	1	11	0	0	8	1.00	1.00	1.00	0.90
L14	0	1	7	0	3	9	0.70	1.00	0.84	0.63
L15	0	0	9	0	2	9	0.82	1.00	0.90	0.80
L16	0	1	8	0	2	9	0.80	1.00	0.89	0.72
L17	2	0	8	2	3	5	0.73	0.71	0.72	0.43
L18	0	0	8	2	3	7	0.73	0.78	0.75	0.50
L20	0	1	11	2	0	6	1.00	0.75	0.89	0.70
L21	3	7	4	2	0	4	1.00	0.67	0.80	0.25
L22	0	1	7	0	3	9	0.70	1.00	0.84	0.63
L23	0	0	5	0	6	9	0.45	1.00	0.70	0.43
L24	0	2	10	0	1	7	0.91	1.00	0.94	0.72
L25	4	0	4	3	5	4	0.44	0.57	0.50	0.02
L27	0	0	11	0	0	9	1.00	1.00	1.00	1.00
L28	0	0	10	7	1	2	0.91	0.22	0.60	0.14
L29	0	2	7	0	3	8	0.70	1.00	0.83	0.55

Missing: Samples with no result due to shipping and/or receiving error–excluded from analysis.

Inconclusive: Samples with inconclusive result as classified by laboratory, unable to determine a positive or negative results. Excluded from the sensitivity and specificity calculations, but included in the kappa calculations.

a: True Positive

b: False Positive

c: False Negative

d: True Negative

Concordance: percent of laboratory results in agreement the CDC

Kappa: **Cohen's kappa coefficient** measures inter-rater agreement considering agreement occurring by chance.

The inter-laboratory agreement by sample is presented in Table 3. The Texas Grey Fox (S01), the Mongoose/Dog Variant (S02), and the Vampire Bat weak positive control (S19), had the lowest level of laboratory concordance with, respectively, only 8/23 (30%), 9/23 (39%) and 9/20 (45%, 3 missing) laboratories correctly identifying the sample. Four of the nine negative samples in the panel had a concordance of less than 0.8, two between 0.8 and 0.9, and only three negative samples had a high level of concordance (0.91, 0.96 and 1.0).

**Table 3:** Agreement between Laboratory and CDC values, by Sample.

Laboratory Results							
Sample	Ag Variant	CDC I/D	Pos	Neg	Indeter-mined	Missing	Concordance (%)
S01	Texas Gray Fox	4+/3+	8	12	3	0	30
S02	Mongoose/ Dog Variant	4+/3+	9	13	1	0	39
S03	Negative	0/0	3	19	1	0	83
S04	AZ Gray Fox (AV7)	3+/4+	18	4	1		83
S05	Negative	0/0	4	16	3	0	74
S06	Lasionycteris noctivagans	4+/4+	19	4	0		87
S07	Tadarida brasiliensis (AV4)	4+/4+	18		0	0	78
S08	Negative	0/0	4	17	2	0	74
S09	Hognose skunk	4+/4+	22	1	0	0	96
S10	South Central Skunk US North Central MX	4+/1+	19	3	1	0	83
S11	Negative	0/0	5	17	1	0	74
S12	Western Eptesicus fuscus	4+/4+	15	8	0	0	65
S13	Negative	0/0	3	15	3	2	76
S14	Negative	0/0	3	19	0	1	86
S15	Negative	0/0	3	20	0	0	91
S16	Mongoose (PuertoRico)	4+/4+	20	2	1	0	91
S17	Negative	0/0	1	22	0	0	96
S18 Control 1	LA Dog Variant	4+/4+	19	2	0	2	95
S19 Control 2	3 (Vampire)	4+/2+	9	7	4	3	45
S20 Control 3	Negative	0/0	0	21	0	2	100

CDC I/D: CDC determined values for Staining Intensity and Antigen Distribution

Pos: Number of laboratories reporting the sample positive for rabies

Neg: Number of laboratories reporting the sample negative for rabies

Inconclusive: Samples with inconclusive result as classified by laboratory, unable to determine a positive or negative results. Excluded from the sensitivity and specificity calculations, but included in the kappa and concordance calculations. Concordance: percent of laboratory results in agreement the CDC

### Logistics and quality control questionnaire

The mean shipping time for laboratories to receive their samples was 11.87 days, ranging from 1 to 65 days (n = 22). Twenty laboratories (87%) reported on the tissue quality of the samples; two of the 20 reported on the quality of samples 1–18, omitting the control sample quality. The mean percentage of samples with deficient microscopic condition and impression quality, as reported by each laboratory, was 13% (range: 0–68%) and 18% (range: 0–63%), respectively. The three control samples (S18-S20) had the highest proportion of samples reported as Deficient (5/18, 5/18 and 7/18, respectively) when considering impression quality. No statistically significant correlation was found between shipping time and the percent of tissues with deficient impression (Pearson’s r = 0.06, p-value = 0.77) or microscopic condition (Pearson’s r = 0.13, p = 0.53, respectively).

### Quality control verification

Four months into the exercise and due to perceived problems with the slides reported by several participating laboratories, we assessed the stability of the rabies antigen and the reproducibility of the DFA test results. Duplicate sets of slides were removed from the -80°C storage at the CDC and tested for stability under different storage conditions and time. The complete panel was maintained under 5 different temperatures (-80°C, -20°C and 4°C, room temperature and 37°C) as well as 3 different storage times (3, 7 and 14 days) to mimic potential transport and storage conditions. All combinations of these conditions were run using two different anti-rabies conjugates (FDI Fujirebio Diagnostics Inc. cat# 800–092 and EMD Millipore Light Diagnostics cat#5100), and one control conjugate EMD Millipore Cat#5102 (non-rabies FITC labeled antibody of the same isotype as the FITC labelled monoclonal) to assess the specificity of the reaction. Both the fluorescent intensity and the antigen distribution were reported for each case. A total of 30 test results (replicas) were reported and analyzed for each of the 20 samples of the panel. The result of this testing was compared against the baseline value provided by the CDC. There were sufficient sample slides for all planned storage variations except sample# 12 (WEF variant).

Results indicate that weak positive control (S19) had false negative results at day 3, 7 and 14 at both -80 and 4°C. Sample 1 also had a negative result after 3 days in storage at -80°C but was positive at other times and storage temperatures. Inconsistent results for these two samples indicate this sample had uneven amount of antigen distributed across the tissue, which is consistent with a low viral load.

### Laboratory practices technical questionnaire

The twenty-three participating laboratories answered the practices questionnaire on laboratory testing, biosafety and quality control (Tables 4 and 5). All laboratories performed DFA and had a dedicated rabies laboratory, which was restricted to authorized personnel in 21 laboratories (91%). The laboratories varied in capacity with 52% testing 0–10 samples per week; one laboratory, affiliated to MoAg reported an average of 120+ samples per week. Only 13 (56%) laboratories responded that the darkroom used for reading DFA slides was for rabies only. While 22 of 23 laboratories reported having a biological safety cabinet, only 65% are checked annually and 74% are for rabies only.

**Table 4:** Selected questions of the rabies laboratory practices questionnaire and results.

Survey Item and Response	Freq.	Percent	Sensitivity(Mean)	Test Stat	p-value
Number of rabies samples tested per week			-	0.07	0.93
0–10	12	52	0.78	-	-
11–30	5	22	0.78	-	-
>30	6	26	0.75	-	-
Number of people performing tests in the lab			-	2.5	0.11
1–2	8	35	0.86	-	-
3–4	11	48	0.69	-	-
4+	4	17	0.81	-	-
Has a formal quality program			-	44.5	0.82
Yes	17	74	0.78	-	-
No	6	26	0.74	-	-
Has a quality control person with direct access to top level management			-	35.5	1.00
Yes	19	83	0.78	-	-
No	4	17	0.75	-	-
Has established, written lab quality manual			-	24.5	0.65
Yes	21	91	0.76	-	-
No	2	9	0.85	-	-
Rabies lab is restricted to the (rabies) testing personnel			-	26.0	0.53
Yes	21	91	0.76	-	-
No	2	9	0.86	-	-
Biological safety cabinet systems annually verified			-	48.5	1.00
Yes	15	65	0.78	-	-
No	7	30	0.76	-	-
Missing	1	4			
Biological safety cabinet restricted for rabies testing			-	25.5	0.24
Yes	17	74	0.80	-	-
No	5	22	0.68	-	-
Missing	1	4			
Has a fluorescence microscope			-	-	-
Yes	23	100	-	-	-
No	0	0	-	-	-
Age of scope (years)			-	1.63	0.22
0–5	10	44	0.80	-	-
6–15	8	35	0.68	-	-
>15	5	22	0.85	-	-
Optical surfaces cleaned after every use			-	44.5	0.49
Yes	18	78	0.76	-	-
No	5	22	0.84	-	-

**Table 5:** Selected questions of the rabies laboratory practices questionnaire and results.

Survey Item and Response	Freq.	Percent	Sensitivity(Mean)	Test Stat	p-value
Service and maintenance records available			-	8.0	0.19
Yes	21	91	0.79	-	-
No	2	9	0.56	-	-
Has calibration programs for the laboratory instruments			-	51.0	0.94
Yes	16	70	0.78	-	-
No	7	30	0.76	-	-
Has an official (institutional) DFA protocol			-	58.5	0.06
Yes	19	83	0.74	-	-
No	4	17	0.92	-	-
Approval and/or rejection criteria of samples established and written			-	25.5	0.39
Yes	4	17	0.85	-	-
No	19	83	0.76	-	-
# of people who read slides per test			-	71.5	0.49
1	11	48	0.75	-	-
2–3	12	52	0.79	-	-
Performs confirmatory test(s) on weak or inconclusive samples			-	46.5	0.09
Yes	20	87	0.74	-	-
No	3	13	0.91	-	-
Participates in a proficiency program for rabies			-	54.0	0.94
Yes	8	35	0.78	-	-
No	15	65	0.77	-	-
Routinely checks VNA titer of all rabies lab employees			-	54.5	0.95
Yes	15	65	0.78	-	-
No	8	35	0.76	-	-
Area of brain used for samples (labs may report >1 area)			-	-	-
Hippocampus	17	74	-	-	-
Cerebellum	16	70	-	-	-
Cortex	11	48	-	-	-
Brain Stem (medulla oblongata)	11	48	-	-	-
Pons	2	9	-	-	-
Thalamus	1	4	-	-	-
Spinal cord	1	4	-	-	-
Olfactory Trigone	1	4	-	-	-
# of Anti-Rabies Conjugates Used			-	5.0	0.11
1	20	87	0.75	-	-
2	3	13	0.96	-	-
Uses rabies negative control FITC labeled Conjugate			-	52.5	0.45
Yes	5	22	0.72	-	-
No	18	78	0.79	-	-
Slide Incubation Time (Minutes)			-	20.5	0.47
30 or less	20	87	0.76	-	-
>30	3	13	0.84	-	-

While all laboratories reported having a fluorescence microscope, the manufacturer brand is highly variable. The age of the microscope varied from less than one year (43%) to greater than 20 years (14%); with 30% of scopes older than 11 years. The light source of the microscope is also highly variable with 82% of the labs using Mercury lamps (HBO 100W or 50W), while only 13% using LED and 4% using halogen as a light source. For routine scanning of the DFA slides, 4 laboratories (17%) use a magnifying power of 40X or higher with oil immersion, 7 laboratories (30%) use a combination of 20X and 40X dry format, and 11 (48%) of the laboratories use 40X in a dry format. One laboratory did not know what type of lens was used to scan the slides. Eighteen (78%) laboratories clean the optical lenses after each use, 21 (91%) have service records and 16 (70%) have calibration programs.

All laboratories reported having written procedures for sample processing, including transportation, reception, handling, protection storage and safe disposal. However, only 19 (82%) laboratories had an official DFA protocol and only 4 (17%) had written sample approval and or rejection criteria.

We found considerable variation in the general laboratory protocols including the area of the brain used for routine diagnosis, the type of sample used (impressions vs. brain smears), the fixation protocol (temperature, time and dilutions for slides) and the conjugate procedures (staining and incubation time). Regarding the selected area of the brain routinely tested, most laboratories selected from more than one location: 17 laboratories (74%) select the hippocampus, 16 (70%) the cerebellum, 11 (48%) the cortex and the 11 (48%) brain stem. In addition, a smaller proportion of the labs select the Pons, Thalamus, Spinal cord or olfactory trigone (n = 3, 13%).

The vast majority of laboratories use brain impressions (n = 22, 96%). Only one laboratory uses brain smears. Nineteen (82%) of the responding laboratories fix the DFA slides. The fixing solution is also variable with 17 (74%) of laboratories fixing in acetone. Of the18 laboratories that reported on fixing times, 13 (72%) employed between 15 to 60 minutes, 4 (22%) from 1 to 4 hrs, and 1 left the slides overnight. Of the 20 laboratories that reported on fixation temperatures, 16 (69%) reported temperatures of -20°C, and the remaining three reported temperatures of 80°C, 4°C, and room temperature, respectively.

The conjugate is a critical reagent in DFA; 20 (87%) laboratories use a single conjugate for rabies diagnosis, while 3 laboratories use more than one. Sixty-five percent of responders using a commercial source and 35% using a conjugated produced by a regional, national or local rabies laboratory. Eighteen (78%) laboratories do not use a rabies negative control FITC labeled conjugate. Eighteen (78%) laboratories incubate the stained slides for 30 min while 5 laboratories incubate the slides for 15, 20, 40, 45 and 60 minutes, respectively.

One laboratory incubates the stained slides at room temperature while the rest of laboratories (22, 95%) incubate at 37°C. Differences were also seen in the solutions used for washing the slides, the time and washing steps and the mounting media used to coverslip: 17% of the laboratories use 2x 3–5 min PBS rinses and 13% use as mounting media 90% glycerol in carbonate-bicarbonate buffer pH 9.0. Eleven (48%) laboratories use only one dedicated slide reader, 10 (43%) use 2 and 2 laboratories have 3 readers per slide. Although 20 (86%) laboratories perform a confirmatory testing on weak or inconclusive results, 3 (13%) do not have this capacity. The most common confirmatory test reported was the mouse inoculation (n = 15, 65%) followed by repeat DFA with using an un-labelled monoclonal to assess the specificity of the fluorescence (n = 4, 17%). Only one laboratory uses cell culture and two laboratories (9%) use RT-PCR.

Sixty-five percent of the laboratories [15] reported having at least three dedicated laboratory employees for rabies diagnostics. While all the laboratories require pre-exposure prophylaxis for their employees, 5 (22%) check the employee titer biannually, 7 (30%) annually, 3 (9%) biennially and 9 (39%) reported that they don’t check or did not have a specific time frame for checking employee titers.

Only 8 laboratories (35%) reported participating in a proficiency testing program for rabies, different from this one, 17 (74%) had a formal quality control program, and 19 (82.6%) had a person responsible for quality control with access to top level management.

## Discussion

The level of concordance between the 23 participating laboratories and the CDC panel showed large variability. Two laboratories had 100% concordance, while 91% of the labs had at least one discordant sample, with a total of 26 false positive and 61 false negative results among all laboratories. This level of concordance is lower than those found in similar inter-laboratory exercises of DFA diagnosis of rabies elsewhere. In an inter-laboratory exercise of Middle and Eastern European countries in 2001 [11], 3 of the 16 (19%) participating national laboratories produced false-positive results. During the EURL/ANSES annual inter-laboratory trials from 2009 to 2014, which included 5 laboratories from the Americas, the percent of laboratories with at least one discordant sample ranged from 8% to 20% [15]. One possible cause of the lower level of concordance relative to other studies, may be related to the sample stability during shipping and conditions of storage of the panel. However, we did not find a significant association between the shipping time and the laboratory-reported microscopic and tissue impression quality. This assumes that shipping time captures adequately unobserved variables such as shipping and storage temperature. At the times and temperatures of shipping and/or storage evaluated in this study, the CDC found no impact on the degree of positivity of samples, with the exception of the weak positive control. Only 4 panels had shipping times longer than the 14 day limit of the stability test (2 labs with 15 and 16 days, respectively). In addition, we tested the level of concordance for the subset of PT panels delivered within the 14-day limit of the stability test, which encompasses 81% of all the panels tested across LAC, and found no significant increase in the concordance among laboratories.

For the current exercise, a technical questionnaire was completed in parallel to the panel in order to identify variations in methodology, equipment and human resources that could potentially explain discordant results to help implement standardized rabies diagnosis protocols across the region. While no significant associations were seen between differences in survey responses and laboratory performance, this may be due to the small sample size, as even small methodological changes have been shown to affect the sensitivity of the DFA test including the mounting medium [16–17], laboratorian’s expertise level, number of employees reading the slides, the anti-rabies conjugate utilized, specificity controls used and working performance of fluorescence microscope [18, 19].

Forty-percent of the laboratories have only one person reading the slides. While no difference in sensitivity was found between laboratories with one or two readers, the OIE manual for DFA recommends two readers per slide [3] and in the EURL/ANSES study, laboratories using two readers had a higher concordance during the DFA than laboratories with one reader [18].

Half of the participating laboratories tested less than 10 samples per week. While the sensitivity did not vary by laboratory work load in this study, laboratories with small throughput may have reduced test interpretation expertise. In the Barrat 2001 inter-laboratory technical questionnaire, low laboratory throughput was generally associated with a lower frequency in the use of fresh (recently prepared, replaced for new one from the bottle or reagents far from expiring) reagents including fixatives, buffers and solutions, which in turn will affect the analytical sensitivity and quality of the test [14].

The type of conjugate may also affect the sensitivity of the DFA test (monoclonal cocktail versus polyclonal, in-house made versus commercial). For the current exercise, laboratories used commercial (65%) or in-house (35%) conjugates. A study of 12 rabies reference laboratories in Europe demonstrated that the variability of conjugates could potentially lead to discordant results and influence assay sensitivity [19]. The OIE manual states that the conjugates should be fully validated for specificity and sensitivity before use, including their ability to detect lyssaviruses other than rabies [3]; it is unclear whether such validation has been completed for the in-house conjugates used in the LAC region. It was recommended by members of the regional laboratory network during a briefing on the results of this exercise that PAHO should provide a standard set of conjugates for future exercises to reduce the potential variability in results associated with conjugate type.

The maintenance, type and quality of fluorescence microscope including quality and type optical lens, magnification and numerical aperture could also affect the DFA results. All labs reported having a fluorescence microscope. However, the manufacturer brand, age, light source and magnifying power was highly variable, which may have affected the overall concordance results among laboratories.

The PT panel was composed of 20 samples with samples of diverse origin. Potentially, the variety of samples and virus variants caused difficulty for some laboratories. The Texas Grey Fox (S01), the Mongoose/Dog Variant (S02), and the Vampire Bat weak positive control (S19) had the lowest level of laboratory concordance. In a two-year review of the 2009–2010 EURL/ANSES international inter-laboratory trials all errors were associated with bat strains (EBLV-1, EBLV-2 and ABLV); the authors commented that these strains do not provide the same type of fluorescence than the conventional RABV strains, which could lead to false negative results [20].

Among the participating laboratories, 28% did not have a written DFA protocol and 82% did not have a written sample approval or rejection criteria. There was variation in the general protocol for sample selection and processing, including the area of brain routinely tested, the type of sample (impressions vs. brain smears), and the fixation protocol, including temperature, time and dilutions for the slides. Details in execution of the immune-staining procedure, including incubation times, incubation temperatures, number of washes as well as mounting medium used also varied greatly. Variations in slide preparation and in the DFA protocols were noted in the 2001 WHO inter-laboratory study [14], particularly in the use of heat and/or acetone for fixation and fixation time and temperature. Variation in methodology including rinsing and washing times, fixation procedures, type of conjugate used and the staining time and temperature was also noted in the 2009–2010 EURL/ANSES technical questionnaires; nevertheless, in this study, the number of persons reading the same slide was the only factor that significantly affected the proficiency test results [18]. Standardized protocols for DFA exist from the OIE Terrestrial Manual, the CDC, the EURL and the WHO [12, 21, 3]. The LAC region would benefit from the use of standardized DFA protocols.

Three of the 23 laboratories did not have the capacity to perform a confirmatory testing on weak or inconclusive results. The most common confirmatory test reported was the mouse inoculation; only two laboratories use RT-PCR. Although, the WHO does not currently recommend RT-PCR as a primary rabies routine diagnostic tool, a number of laboratories in LAC are in the process of implementing RT-PCR as a confirmatory test as ascertained during the post-exercise briefing. In an European ring test study, RT-PCR showed a high level of variation between laboratories with cross-contamination as a potential issue [4]. Concerns about the potential of RT-PCR to identify imported strains were raised by Fischer et al., 2013. Given the expressed interest of LAC laboratories to implement RT-PCR, a region-wide RT-PCR harmonization exercise is recommended due to the test’s requirements for strict quality control protocols and high level of experience and expertise for accurate diagnosis [2].

In contrast to the acetone-fixed slides provided in this exercise, the three inter-laboratory studies cited in this discussion used freeze-dried viable virus homogenates obtained from animals inoculated intra-cerebrally [14, 15, 20]. The use of fully inactivated gamma radiated homogenates in future iterations of this exercise would also allow for each lab to test its slide or smear preparation protocol as commented in Barrat et al.,2007. Furthermore, homogenizing the material in their own laboratory settings may provide more consistency across the panels than impressions from an infected brain [14].

The WHO recommends the preventive immunization of all staff handling infected or suspect material with the titer checked every 6 months. While all of the laboratories participating in this exercise required pre-exposure prophylaxis for employees, only 21.7% of the labs reported that employee titers are checked in compliance of WHO-recommendations. Employee safety should be a primary concern for all rabies laboratories and a regional effort to increase the frequency of employee titer checks should be implemented.

As a first iteration of a regional DFA inter-laboratory exercise, this effort provided useful insights into the lab performance and protocols, as well as suggested improvements for future rounds of the exercise. There is a clear need to increase participation in regional exercises on rabies diagnosis across LAC, since only 34.8% of the 23 laboratories reported previous participation in diagnostic test exercises of this nature. A regional laboratory network (REDILAR) is part of the REDIPRA plan for elimination of canine-rabies in the Americas [6]. Noting the heterogeneity in the DFA protocols described in the technical surveys conducted in this study, and the level of discordant results, an effective REDILAR network is critical to develop a rabies diagnosis and reporting harmonization scheme concerted with an annual testing exercise in the way it has been implemented for Europe [21, 22].

Results of this exercise were presented before the Regional Meeting of the Rabies Directors of the Americas in 2015 (REDIPRA 15), which end up in an official resolution instructing the REDILAR coordinated by PANAFTOSA, to define a framework for the harmonization of rabies diagnosis and to implement annual exercises. This resolution demonstrates the commitment by the national authorities, PAHO, and WHO Collaborating Centers for Rabies Research to support the REDILAR laboratory network in improving the sensitivity of rabies diagnosis. Sensitivity, specificity as well as timely rabies diagnosis and surveillance will be of increasing relevance in the race towards elimination of human rabies transmitted by dogs in the Region.

## Supporting information

S1 Table Results of the Rabies Panel Testing by laboratory and sample. (XLSX)

S2 Table Responses to the laboratory practices assessment by laboratory. (XLSX)
